# Short and long-term effects of exergaming for the elderly

**DOI:** 10.1186/s40064-016-2379-y

**Published:** 2016-06-21

**Authors:** Yasunori Nagano, Kenji Ishida, Toshikazu Tani, Motohiro Kawasaki, Masahiko Ikeuchi

**Affiliations:** Department of Rehabilitation Center, Kochi Medical School Hospital, Nankoku, Kochi 783-8505 Japan; Department of Rehabilitation, Kurihara Central Hospital, Kurihara, Miyagi 987-2205 Japan; Department of Orthopaedic Surgery, Kubokawa Hospital, Takaokagun Shimantocho, Kochi 786-0002 Japan; Department of Orthopaedic Surgery, Kochi Medical School, Nankoku, Kochi 783-8505 Japan

**Keywords:** Exergaming, Step training, Exercise continuation

## Abstract

**Objective:**

Exergaming has been introduced in safe and beneficial intervention for the elderly. However, no study has examined exergaming-based interventions for the elderly that last several years. Therefore, we investigated the effectiveness and safety of a 12-week intervention using step training with exergaming for the elderly (12-week study). Moreover, we conducted an exergaming-based intervention for 3 years (3-year study).

**Materials and methods:**

12-week study: Forty-two elderly persons participated in this study. Using an in-house developed exergaming protocol, a step training was performed for 15 min/session twice a week for 12 weeks. We investigated post-intervention changes in motor functions, successful step-rate, the intensity of exercise, which was evaluated using Borg scale (Rating of Perceived Exertion). 3-year study: An intervention using exergaming was conducted for 12 weeks by 20 elderly participants. Two courses/year of exercise were performed at 3-month intervals. This was continued for 3 years. The exercise continuation rate, its influence on motor functions were investigated.

**Results:**

12-week study: Lower-limb muscle strength, balancing capacity, and whole body reaction time significantly improved in the exercise group. The mean score on Borg scale was 12 ± 2 on the first day of the step exercise and 9 ± 2 on the final day. 3-year study: Exercise was continued in 16 out of the 20 subjects. The Timed Up and Go Test, duration of one-footed standing, and quadriceps strength significantly improved.

**Conclusion:**

The results of the present study showed that exergaming provided an appropriate exercise intensity for the elderly and safely improved motor functions. The exercise continuation rate in the 3-year study was high. Improvements in motor functions may contribute to the prevention of falls.

## Background

The rapid aging of society has recently become more prominent in first world countries. In order to prolong not only average life spans, but also healthy life expectancy, it is important to maintain and/or improve motor functions. Since muscle strength and balancing capacity decrease with age, the risk of falls may be avoided by exercise interventions that improve these functions. A systematic review presented by Sherrington et al. (2008) revealed that complex training, involving various types of muscle strength-potentiating and balance-maintaining exercises, decreased the risk of falls.

Muscle strength around the ankle and hip joints is weaker in the elderly than in the young (Bemben et al. 1996; Kirkendall and Garrett Jr 1998; Doherty 2003; Janssen et al. 2000). In the likelihood of a fall, muscles around the ankle and hip joints in the young respond in order to prevent the fall (ankle/hip strategies) (Manchester et al. 1989; Woollacott 1993; Mackey and Robinovitch 2006). On the other hand in the elderly, stepping predominantly contributes to the prevention of falls (stepping strategy). Because the ankle/hip strategy-dependent prevention of falls is compromised, if the elderly cannot prevent falls through the stepping strategy, proximal femoral fractures may occur (McIlroy and Maki 1996; Maki and McIlroy 2006; Luchies et al. 1994; Rogers and Mille 2003). Among elderly-specific fractures, proximal femoral fractures have the strongest impact on healthy life expectancy and prognosis. Therefore, reinforcing lateral stability is important for preventing these fractures (Greenspan et al. 1998; Aizen et al. 2003; Hwang et al. 2011). Repetitive step training is useful for improving the lateral stability of subjects (Maki and McIlroy 2005).

Repetitive step exercises are monotonous, which may reduce compliance (Oman and King 2000; Dishman and Buckworth 1996). Exercise training using a computer game (exergaming) was developed as a compliance-improving training method in order for training to become more enjoyable (Warburton et al. 2007; Tamura et al. 2007; Baranowski et al. 2008). The positive effects of exergaming, involving repetitive step exercises, have been reported in elementary school children, healthy young adults, and obese adults (Taylor et al. 2011; Bailey and McInnis 2011; Sell et al. 2008). The safety, efficacy and lower-limb motor functional changes of exergaming for several months have been reported in the elderly (Skjæret et al. 2015a). However, the exercise continuation rate, its safety, and its influence on motor functions in the elderly periodically subjected to interventions with exergaming for several years have not yet been examined.

Since the game speed in commercially available computer games is fast, it is difficult for the elderly to acquire a high score (Clark and Kraemer 2009; Skjæret et al. 2015a, b). Therefore, the degree of difficulty in exergaming needs to be regulated so that the elderly may enjoy it (Smith et al. 2011). Smith et al. investigated the success rate of stepping in the elderly using a stepping device that was developed based on a conventional computer game. They indicated that the successful step-rate was 100 % at a pace of 1 step per 2 s. When the degree of difficulty was changed, the success rates of stepping were approximately 80 % at a pace of 1 step/s and approximately 66 % at a pace of 1.25 steps/s (Smith et al. 2011). A commercially available stepping game, Dance Dance Revolution (DDR, Konami), initially provides a stepping pace of approximately 1 step/s, and this is gradually accelerates as the game progresses, ultimately reaching approximately 3 steps/s. This pace is too fast for the elderly (Smith et al. 2011); therefore, the degree of difficulty needs to be regulated.

We had to develop software for step training program, in which the degree of difficulty was regulated. We had to develop software for a step-training program, in which the degree of difficulty was regulated. When we developed the software, there had been only one previous report that had investigated the relationship between successful steps and the step-rate of exergaming for the elderly. (Smith et al. 2011). Therefore, we referred to this report and regulated the step-rate of the training program accordingly. In this software, the step-rate was established as 1 step/s (Smith et al. 2011), a pace at which the elderly have the potential to acquire a high score.

Past researchers have reported that adults start with easier games and progress to more difficult games (Baranes et al. 2014). Therefore, clearly the difficulty level of exergaming should be set at a low level when setting up step training. In order to improve their motivation to continue exercising, we prepared a local festival (Yosakoi Festival) model as the game design. Residents in the area are familiar with this festival. The model, with a musical instrument as the symbol of the Yosakoi Festival, was set to move at every step so that everyone was capable of enjoying. The participants recorded their results in their notebooks after every session. They were also able to compare them with previous scores. We utilized their notebooks to maintain their motivation to continue exercising.

The aim of this study was to verify our hypotheses: 1. Exergaming, in which the degree of difficulty was regulated, is safe for healthy individuals, has the high successful step-rate of the training and it is effective for improving lower-limb motor functions; and 2. Periodic interventions with exergaming for several years are safe with a high continuation rate (attendance rate, number of participants) and are useful for improving motor functions.

## Methods

### Participants

#### 12-week study

Forty-two healthy elderly persons [28 males, 14 females, mean age: 71 ± 5 years (64–85 years)] participated in this study. This group of subjects consisted of very healthy elderly citizens paid a regular salary for physical labor. Therefore, their motor function ability was better than the average elderly citizen. The criteria for participation included an age of 60 years or older, orthosis-free walking, and a healthy status without exercise restrictions. Exclusion criteria included a history of lower limb surgery (fracture/artificial joint), history of cerebrovascular disorders, diabetes, neurodegenerative diseases (such as Parkinson’s disease) that cause balance disturbances, exercise-affecting diseases (heart/lung/kidney diseases), numbness/sensory disturbances in the hands/legs, and dementia. Institutional review board approval was obtained prior to the study. All subjects agreed in writing to participate in the study after reading and signing an informed consent form. They were randomly allocated to the exercise or control group. Randomization was performed by using a list of random numbers and a sealed envelope method. Participants were divided into two groups: exercise and control groups, based on their agreement with random assignment in order to achieve a male-to-female ratio of 1:1. Their daily activities remained unchanged during the study period. Eight out of the 42 subjects had a history of falls in the past year (exercise group: 5, control group: 3).

#### 3-year study

Subjects were 20 elderly residents in a fishery town (population: approximately 12,000, proportion of elderly persons: approximately 39 %) who wanted to participate in this study. This group of subjects consisted of frail elderly persons who scored low on motor function in a musculoskeletal function examination carried out in a residential area. They consisted of 4 males and 16 females, with a mean age of 78 ± 2 years. Exclusion criteria included exercise-affecting diseases (heart/lung/kidney diseases), neurodegenerative diseases (such as Parkinson’s disease) that cause balance disturbances, dementia, and gait disorders (hemiplegia after cerebral infarction and neurodegenerative diseases).

### Step exergaming program

**Fig. 1:**
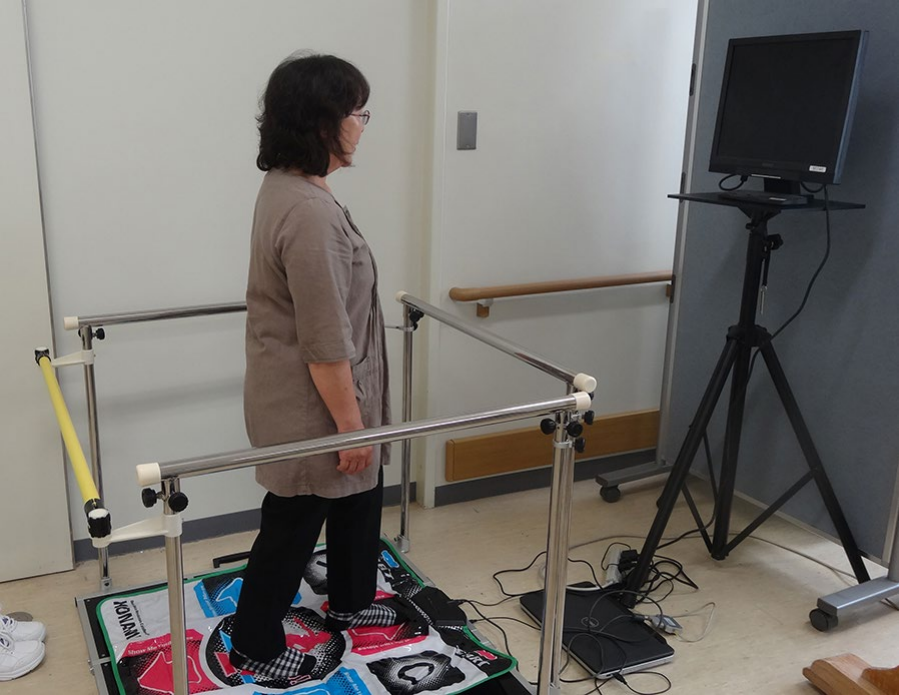
Appearance of the newly developed exergaming program

**Fig. 2:**
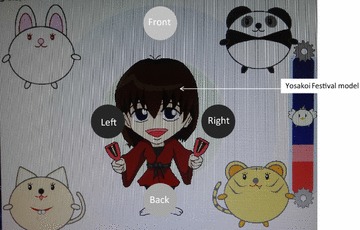
Game monitor

**Fig. 3:**
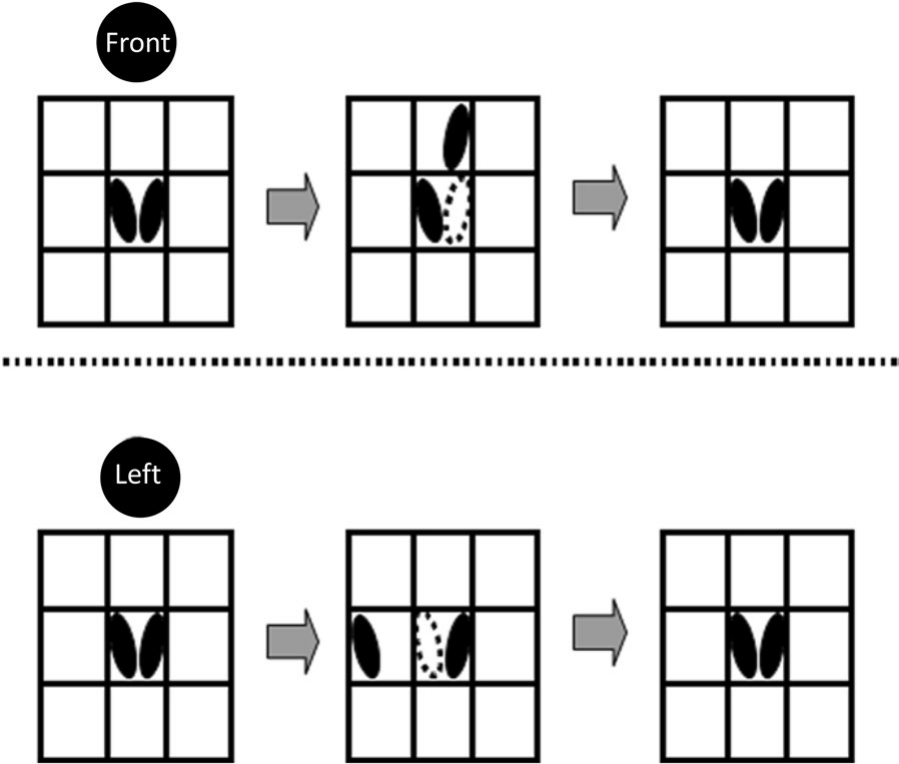
Scheme of the step training

We developed software for a step training program, and used it (exergaming) in the exercise group. The system consisted of a stepping mat that was connected to a universal serial bus (USB), a 19-inch display, and a uniquely prepared program (Fig. 1). The mat for stepping measured approximately 1 m^2^, and the distance between the center of the mat and the display was approximately 1.5 m. The game contents were as follows: 4 yellow disks, entitled “Front”, “Back”, “Left”, and “Right”, randomly appeared from the outer boundary of the display, and were slowly transferred toward the center of the display. The disk color changed from yellow to red, and disappeared after 1 s. If one leg was stepped in the direction indicated by the disk within 1 s from red coloration until its disappearance, the subject got a score. The model had a musical instrument as the symbol of the Yosakoi Festival (Fig. 2). It moved the musical instrument with each score; therefore, increasing the likelihood that each subject would enjoy the exercise. Upper row: If the “Front” disk was transferred from the upper area of the monitor toward its center, one leg had to step out in the front direction before the disk disappeared. The stepping leg was then returned to the center. In this study, we did not decide whether to use the same foot in the front step or not. Lower row: If the “Left” disk was transferred from the left side of the monitor toward its center, the left leg had to step out in the left direction before the disk disappeared. The stepping leg was then returned to the center (Fig. 3).

The step-rate based on disk indications was established as 1 step/s. The total number of steps per training session when a full score was achieved was established as 672 so that the frequencies of the 4 steps, “Front”, “Back”, “Left”, and “Right”, were similar.

One training session was completed after both successful and unsuccessful steps were counted towards the 672 steps required.

At the completion of each session of the step training, the successful step-rate (expressed as %) was presented to each subject in order to improve his/her motivation to increase it.

### Exergaming protocol

#### 12-week study

Exergaming for 15 min/session was performed twice a week for 12 weeks. Exercise activities were conducted in the Department of Rehabilitation of our hospital. Subjects were instructed to record their successful step-rate in their notebooks. We confirmed the presence or absence of adverse events during exercise in each participant at every session.

#### 3-year study

Exergaming for 15 min/session was performed twice a week for 12 weeks (1 course). After a 3-month interval (no step training), it was similarly conducted (2 courses per year). This was repeated for 3 years. Exercise activities were conducted in the health facility of the area. Public health nurses confirmed the number of participants and the presence or absence of adverse events during exercise in every session.

### Outcome measures

Motor function-evaluating items

1. 10-m walking test (s)

This test was carried out according to the reference (Novaes et al. 2011). Three trials were performed and the average value was reported.

(2) TUG (s)

This test was conducted using the method described by Shumway-Cook et al. (2000) and Podsiadlo and Richardson (1991). Subjects were instructed to sit deeply in a chair with a height allowing the hip and knees to flex at 90°, with their back against the back rest.

Subjects were instructed that their backs must touch very lightly on the back rest.

Stable posture was confirmed by the examiner. They stood up with a “GO” sign, walked at a comfortable speed, and turned back at a cone placed 3 m ahead as a mark. The interval until they sat in the chair again was measured 3 times, and the mean value was adopted.

(3) Tandem gait test (step)

Subjects were instructed to walk with bare feet on a straight line at an arbitrary speed such that the toes of the back foot touched the heel of the front foot at each step. The number of tandem gait steps was measured 3 times, and the maximum was adopted.

(4) Duration of one-footed standing (s)

Using a stopwatch, the time for which subjects were capable of maintaining a one-footed standing position (Bohannon et al. 1984) (with the eyes opened and bare feet) was measured 3 times per side, and the maximum was adopted. Subjects were instructed to raise one leg 2–3 cm from the floor. Measurements were started when the posture became stable a few seconds after the start of one-footed standing. Stable posture was confirmed by the examiner. It was completed when the raised leg reached the ground, when it touched the axis leg, when the axis leg’s position moved, or after 60 s or more.

(5) Measurement of quadriceps/gluteus medius muscle strength (N)

Muscle strength was evaluated using a hand-held dynamometer (ANIMA, μTas F-1). Quadriceps/gluteus medius muscle strength was measured in line with the same measurement procedures used in two previous studies (Koblbauer et al. 2011; Krause et al. 2007). The strength of each muscle was measured 3 times on the left and right sides, and the mean value was adopted. Muscle strength measurements using a hand-held dynamometer were performed by the same examiner for each muscle.

(6) Platform stabilometry (cm)

Using a platform stabilometer (Gravicorder, ANIMA GS3000, Tokyo, Japan), the locus length per unit area (cm) on eye-opening was evaluated (Kapteyn et al. 1983).

(7) Pole drop test (cm)

A pole graduated in centimeters, 2 cm in diameter, 55 cm in length, and 115 g in weight (TKK 5008, Takei Corp, Tokyo, Japan), was held by the examiner with the 0 cm line between, but not touching, the subject’s thumb and fingers. When instructed, the subject caught the pole as quickly as possible between his or her thumb and fingers immediately after the examiner released it without warning. Three trials were performed and the mean value of the centimeter marks at which the subject caught the pole was reported. Three trials were performed and the best value was reported.

(8) Whole body reaction time (WBRT) (s)

The ability to promptly convert visual information to lower-limb exercise (agility) was measured using a TKK5408 device (Takei Corp, Tokyo, Japan). Subjects were instructed to stand on a sensor mat and jump on seeing randomly lighting flashes in front (Miyatake et al. 2012). The interval until both feet completely separated from the mat was measured 5 times, and the mean value was adopted.

#### 12-week study

##### Motor function assessment

In the exercise and control groups, evaluation items (1)–(8) were measured before the start of the intervention and after its completion. Physical and occupational therapists were responsible for taking measurements.

##### Successful step-rate, intensity of the exercise

In the exercise group, we had recorded the successful step-rate. Borg scale was used to determine whether the intensity of the exercise was adequate for the elderly in the first and final exercise sessions. Borg scale has been reported as Rating of Perceived Exertion (RPE) (Borg 1982).

##### Adverse events

Before and after all exercise activities, the presence or absence of adverse events were investigated during the intervention period. Survey items consisted of high blood pressure, falls, and exercise-related pain.

#### 3-year study

##### Exercise continuation rate [attendance rate (%), number of participants (n) in 1-, 2-, 3-year]

The number of participants who discontinued exercise was determined, and the exercise continuation rate was calculated.

##### Motor function assessment

Evaluation items (2), (4), and (5) were measured before the start of the intervention and after 3 years (after completion of the intervention).

##### Adverse events

Before and after all exercise activities, public health nurses investigated the presence or absence of adverse events during the intervention period. Survey items consisted of high blood pressure, falls, and exercise-related pain.

### Statistical analysis

We used the Mann–Whitney U test for evaluating unpaired data, Wilcoxon’s signed rank test for paired data. We used the Friedman’s Test to analyze the attendance rate between the three groups.Categorical variables were compared using the Chi square test. Values are given as mean ± SD. Values were considered significant at P < 0.05. All statistical analyses were performed using SPSS 16.0J (IBM, Chicago, state of Illinois).

### Results

#### 12-week study

##### Demographic and baseline data (Table 1)

In this study, 1 subject in the exercise group developed a respiratory disease during the training period, leading to the discontinuation of exercise. Pre-intervention assessments were not conducted in 2 subjects in the control group. Therefore, 20 subjects in the exercise group (14 males, 6 females, mean age: 72 ± 5 years) and 19 in the control group (13 males, 6 females, mean age: 72 ± 5 years) were evaluated. No significant differences were observed in any item according to the data obtained for physical characteristics and baseline data for motor functions.

**Table 1:** Comparison in demographic and baseline data between the exercise and the control groups

	Exercise group (n = 20)	Control group (n = 19)	P value
Sex (M/F)	14/6	13/6	0.92^+^
Age (years)	72 ± 5	72 ± 5	0.88
Height (m)	1.58 ± 0.08	1.60 ± 0.09	0.62
Weight (kg)	59.2 ± 8.0	59.3 ± 9.3	0.91
Body mass index (kg/m^2^)	23.8 ± 2.5	23.2 ± 2.9	0.66
Body fat percentage (%)	24.9 ± 7.1	25.1 ± 7.0	0.68
10-m walking test (s)	7.5 ± 0.9	7.0 ± 0.7	0.1
Timed Up and Go test (s)	8.8 ± 1.3	8.1 ± 1.2	0.15
Tandem gait test (step)	9 ± 2	9 ± 2	0.99
One-footed standing duration (s)	46.1 ± 17.8	52.6 ± 13.7	0.31
Strength of quadriceps muscles (N)	249 ± 74	275 ± 109	0.75
Strength of gluteus medius muscles (N)	109 ± 32	109 ± 23	0.73
Pole drop test (cm)	26.9 ± 6.2	27.7 ± 4.9	0.1
Whole body reaction time (s)	0.44 ± 0.06	0.42 ± 0.05	0.26
Platform stabilometry (cm)	23.9 ± 11.1	24.5 ± 7.6	0.59

Values are mean ± SD except sex

P values are given by either by the Chi square test^+^ the Mann–Whitney U test

* Values were considered significant at P < 0.05

##### Motor function assessment (Table 2)

The results obtained for various measurement items before the start of the exercise and at its completion in the exercise and control groups are presented. No significant changes were observed in the control group. The exercise group achieved significant improvements in TUG, the duration of one-footed standing, quadriceps muscle strength, gluteus medius muscle strength, WBRT, and locus length per unit area on platform stabilometry. No significant changes were observed in the 10-m walking, tandem gait and pole drop tests.

**Table 2:** Changes in physical performance at baseline and 12-week measurement

	Exercise group (n = 20)			Control group (n = 19)		
	Baseline	12-Week	P value	Baseline	12-Week	P value
10-m walking test (s)	7.5 ± 0.9	7.3 ± 0.8	0.31	7.0 ± 0.7	7.1 ± 1.0	0.94
Timed Up and Go test (s)	8.8 ± 1.3	8.2 ± 1.0	0.04*	8.1 ± 1.2	8.0 ± 1.4	0.97
Tandem gait test (step)	8.8 ± 2.3	8.9 ± 2.1	0.79	8.7 ± 2.1	8.8 ± 2.0	0.32
One-footed standing duration (s)	46.1 ± 17.8	53.6 ± 12.4	0.04*	52.6 ± 13.7	54.8 ± 13.3	0.46
Strength of quadriceps muscles (N)	249 ± 74	339 ± 85	<0.01*	275 ± 109	276 ± 49	0.88
Strength of gluteus medius muscles (N)	109 ± 32	129 ± 24	<0.01*	109 ± 23	113 ± 34	0.33
Pole drop test (cm)	26.9 ± 6.2	28.4 ± 6.3	0.16	27.7 ± 4.9	29.4 ± 3.3	0.51
Whole body reaction time (s)	0.44 ± 0.06	0.40 ± 0.07	0.01*	0.42 ± 0.05	0.41 ± 0.05	0.43
Platform stabilometry (cm)	23.9 ± 11.1	27.5 ± 9.3	0.04*	24.5 ± 7.6	25.4 ± 8.0	0.66

Values are mean ± SD

P values are given by the Wilcoxon’s signed rank test

* Values were considered significant at P < 0.05

##### Success rate of step (Fig. 4), intensity of the exercise

The mean step-rate was 77.2 % on the first day of the step exercise and 90.4 % on the final day, showing a significant increase (P < 0.05).

**Fig. 4:**
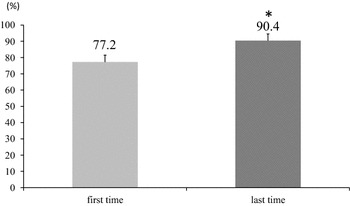
Success rate of step (%) at first, last time (exercise group, 12-week). P values are given by the Wilcoxon’s signed rank test. Values were considered significant at P < 0.05*

The mean score on Borg’s scale was 12 ± 2 on the first day of the step exercise and 9 ± 2 on the final day, showing a significant decrease (P < 0.001). Therefore, an improvement in exercise tolerance was achieved.

##### Adverse events

No adverse events were observed during the 12-week training period.

#### 3-year study

##### Continuation of exercise [attendance rate (%), number of participants (n) at 1-, 2-, 3-year] (Fig. 5)

The attendance rate (%) was 79 % in the 1-year group, 82.6 % in the 2-year group, and 82.4 % in the 3-year group. These changes were not significant. These changes were not significant. The number of participants (n) was 20 in the 1-year group, 17 in the 2-year group, and 16 in the 3-year group. Exercise was discontinued for health-related reasons in 2 subjects and family care in 1. The reason for discontinuation was unclear in another subject. Exercise was continued in 16 out of the 20 subjects.

**Fig. 5:**
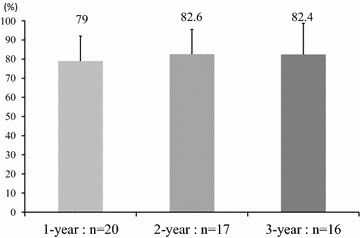
Attendance rate (%), number of participants (n) (1-, 2-, 3-year). P values are given by the Friedman’s test. Values were considered significant at P < 0.05*

##### Motor function assessment (Table 3)

In elderly subjects who were able to continue exercise, motor functions before starting the exercise were compared with those after 3 years (at completion of the exercise). Significant improvements were observed in TUG, the duration of one-footed standing, and lower-limb muscle strength.

**Table 3:** Changes in physical performance at baseline and 3-year measurement (exercise group: n = 16)

	Baseline	3-Year	P value
Timed Up and Go test (s)	10.7 ± 2.4	9.5 ± 2.7	0.03*
One-footed standing duration (s)	23.2 ± 10.3	39.6 ± 21.3	<0.01*
Strength of quadriceps muscles (N)	137 ± 59	179 ± 66	0.02*

Values are mean ± SD

P values are given by the Wilcoxon’s signed rank test

* Values were considered significant at P < 0.05

##### Adverse events

No adverse events were noted during the 3-year training period.

## Discussion

The safety, efficacy and lower-limb motor functional changes of exergaming for several months have been reported in the elderly (Skjæret et al. 2015a). However, exercise continuation rates or changes in motor functions in elderly people subjected to periodic interventions with exergaming for several years have not yet been examined. In the exercise group involving a 12-week intervention, lower-limb muscle strength parameters, quadriceps and gluteus medius muscle strength, and balance parameters, the duration of one-footed standing and platform stabilometry findings, significantly improved. A parameter of lower-limb agility, the WBRT, also significantly improved. These results were attributed to exergaming, which required unexpected multi-directional stepping; the body’s center of gravity was transferred to the front, back, left, or right with every step, and loading to the lower limbs with every step may have improved quadriceps and gluteus medius muscle strength. The frequent transfer of the center of gravity may have simultaneously played the role of balance training. Furthermore, it was necessary to promptly and repeatedly step in the correct direction based on an instant evaluation according to the disk’s indications on the monitor; this may have contributed to improving the agility of the lower limbs. No significant differences were observed in the 10-m walking or tandem gait tests, which may have been because the baseline values before the start of exercise training were approximate to the upper limit of the normal range.

Motor function in the 12-week and 3-year exercise group was significantly different. The 12-week group consisted of very healthy elderly citizens who receive a regular salary for physical labor. Therefore, their motor function ability is better than the average elderly citizen. We think that they were able to stand on one foot longer than the average elderly citizen. Those selected for the 3-year group consisted of elderly citizens who scored low on motor function ability in the musculoskeletal function examination carried out in a residential area. In addition, members of the 3-year group were, on average, older than members of the 12-week group. There was a significantly higher proportion of women in the 3-year group than in the 12-week group. For these reasons, we believe that there is a significant difference in motor function between the two groups.

Out of the four reports on step exergaming in the elderly, only one report had described the step-rate. The step-rate that had been adapted to the elderly was a lower-step rate (0.5–1 step/s). However, this report did not describe how successful the step-rate was (Schoene et al. 2013). In this study, the step-rate was established as 1 step/s. The mean success rate of this step was 77.2 % on the first day of the step exercise, which is similar to the previous report’s finding (Smith et al. 2011). The mean success rate of this step was 90.4 % on the final day, showing a significant increase (P < 0.05). It is possible that this was caused by an improvement in lower limb muscle strength, balance function (how long subjects could stand on one foot and platform stabilometry) and whole body reaction time, which is an indicator of agility. We think that the lower-step program has two benefits. First, it has a highly successful step-rate. Secondly, Watanabe et al. suggested that slow speed exercise is more effective than normal speed exercise (Watanabe et al. 2015). Therefore, a lower-step program may be more effective than a higher-step one.

The results of this study involving a 12-week intervention showed that the mean score on Borg scale at the start of the exercise was 12, which confirmed that the intensity of the exercise was appropriate for the elderly. The mean score was 9 at completion of the exercise. The results showed that the exercise tolerance improved. The mean score on Borg scale at the start of the exercise was 12, which was appropriate as an aerobic exercise for the elderly. There was no excessive exercise loading and no adverse events, such as falls or pain, during the intervention. This result was similar to previous reports (Skjæret et al. 2015a, b).Therefore, exergaming was considered to be safe.

Previous studies reported the efficacy of exergaming using conventional game machines, such as DDR and Nintendo Wii (Taylor et al. 2011; Bailey and McInnis 2011; Sell et al. 2008). These reports were not intended for the elderly. In integrative review (Skjæret et al. 2015a, b), there are four reports of step exergaming in the elderly. Two reports (Pichierri et al. 2012a, b) were a study of cognitive-motor function, which are not relevant to the focus of our study. Another report (Studenski et al. 2010) did not show a detailed motor function evaluation. Schoene et al. (2013) conducted an intervention with DDR 2 or 3 times a week for 8 weeks in 15 elderly people, with a mean age of 78 years. They reported that the interval until stepping to the front, back, left, or right was shortened, suggesting that this program was useful for preventing falls. In their study, no increase was observed in knee-extending muscle strength and TUG, which was different from our results. This may have been because the intervention period in their study (8 weeks) was shorter than in our study (12 weeks). Muscular weakness in the lower limbs is the most important risk factor for falls (American Geriatrics Society, British Geriatrics Society, and American Academy of Orthopaedic Surgeons Panel on Falls Prevention 2001). Exergaming, which improved lower-limb muscle strength, resulted in successful outcomes.

Regarding the effects of interventions with exergaming, although short-term (6–12 weeks) interventions have been reported (Taylor et al. 2011; Bailey and McInnis 2011; Sell et al. 2008; Maloney et al. 2008; Anderson-Hanley et al. 2011; Lamoth et al. 2011; Natbony et al. 2013; Skjæret et al. 2015a, b), no study has conducted an intervention that lasted for several years. Annesi previously reported the dropout rate of a new exercise program started in a public exercise facility (Annesi 2007). The exercise continuation rate a few months after starting the exercise was relatively low (Annesi 2007). We performed an intervention with exergaming for 3 years, and exercise was continued by 16 out of the 20 subjects, showing a high continuation rate. The attendance rate (%) was high (79 % in the 1-year group, 82.6 % in the 2-year group, 82.4 % in the 3-year group). These changes were not significant. A possible reason for the high attendance rate is a report that exergaming increases exercise compliance (Warburton et al. 2007; Tamura et al. 2007; Baranowski et al. 2008). In addition, Motor functions also significantly improved. This may have been related to adequate exercise loading and enjoyable game features. This is the first study on the exergaming continuation rate and its influence on motor functions during a long-term intervention for the elderly. Exergaming was safe and useful for maintaining/improving motor functions.

This study had several limitations. It involved healthy elderly people; high-risk elderly people with a history of frequent falls were not included. Furthermore, subjects in the twelve-week study were arbitrarily divided into exercise and control groups. If a cross-over trial had been conducted, the results obtained may have been more accurate.

In this study, we did not decide whether to use the same foot in the front step or not. Although it often appeared that front stepping was conducted with the right foot, the correct proportions have not been evaluated. In the previous four reports on the elderly (Pichierri et al. 2012a, b; Studenski et al. 2010; Schoene et al. 2013), there was no mention of whether subjects were front stepping with one or both feet, a point left open to debate.

For step exergaming we set the step rate at a constant 1 step/s. A benefit of establishing a constant step rate is that the elderly can obtain a higher score. Since we have not investigated other step-rates, the most effective step-rate for the elderly is open to question. It has been reported that adults start with easier games and progress to more difficult games (Baranes et al. 2014). In order to maintain motivation over the long term, researchers should consider creating a program which gradually increases the degree of difficulty. There is room for further investigation.

In the three-year study, there was no comparison with an age-/sex-matched control group. In addition, the number of subjects was relatively small. A larger sample size may be more representative. The exercise parameters, such as the duration and frequency most effective on lateral instability, remain to be elucidated. Nevertheless, this study has provided a basis for further studies on the preventative effects of a step exercise on falls in the elderly.

## Conclusion

Exergaming was useful for safely improving the motor functions of the elderly, including lower-limb muscle strength, at an adequate intensity of exercise in a short period. In addition, the 3-year intervention confirmed its safety and had a high exercise continuation rate. Furthermore, improvements were observed in motor functions, suggesting the usefulness of exergaming for the prevention of falls in the elderly. A large-scale study needs to be conducted in the future in order to more concretely determine whether exergaming prevents falls.
